# Stem Cell Therapies: A Way to Promising Cures

**DOI:** 10.7759/cureus.5712

**Published:** 2019-09-20

**Authors:** Khalid Nawab, Deepak Bhere, Anthony Bommarito, Muhammad Mufti, Awais Naeem

**Affiliations:** 1 Hospitalist, Geisinger Holy Spirit, Camp Hill, USA; 2 Center for Stem Cell Therapeutics and Imaging, Brigham and Women's Hospital, Boston, USA; 3 Internal Medicine, St. Mary's Medical Center, Long Beach, USA; 4 Internal Medicine, Khyber Teaching Hospital, Peshawar, PAK

**Keywords:** stem cells, oncology, regenerative medicine, cell based therapy, immunotherapy, stem cell transplantation, mesenchymal stem cell, hematology, naive stem cells, engineered stem cells

## Abstract

Stem cells carry the remarkable ability to differentiate into different cell types while retaining the capability to self-replicate and maintain the characteristics of their parent cells, referred to as potency. Stem cells have been studied extensively to better understand human development and organogenesis. Because of advances in stem cell-based therapies, regenerative medicine has seen significant growth. Ophthalmic conditions, some of which are leading causes of blindness worldwide, are being treated with stem cell therapies. Great results have also been obtained in the treatment of oral and maxillofacial defects. Stem-cell-based therapies have great potential in the treatment of chronic medical conditions like diabetes and cardiomyopathy. The unique property of stem cells to migrate towards cancer cells makes them excellent vectors for the transportation of bioactive agents or for targeting cancer cells, both primary and metastatic.

While these therapeutic strategies are extremely promising, they are not without limitations. Failure to completely eradicate the tumor and tumor relapse are some of those concerns. Stem cells share some characteristics with cancer stem cells, raising concerns for increasing the risk of cancer occurrence. Ethical concerns due to the fetal origin of stem cells and cost are other major obstacles in the large-scale implementation of such therapies.

## Introduction and background

**Figure 1:**
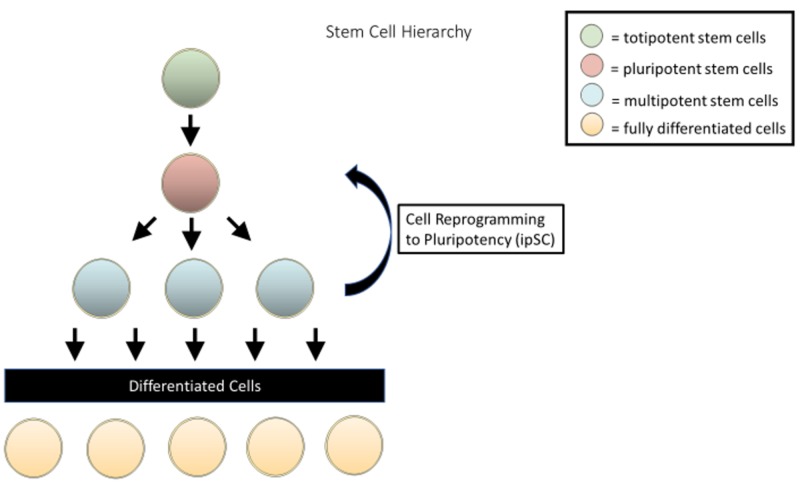
Various stages of stem cell differentiation

Stems cells are characterized by their ability to differentiate into different cell types while retaining the capability to self-replicate and maintain the characteristics of the parent cells [1]. This varying ability of stem cells to differentiate into specialized cell types is referred to as potency. Based on their potency, various types of stem cells are shown in Figure 1. In the spectrum of cell potency, cells that can divide and differentiate into any embryonic cell type, as well as extraembryonic cells, are referred to as totipotent. In the model of human development, totipotent cells arise from zygotes, which are single totipotent cells. As the zygote divides in the days following fertilization, the identical daughter cells remain totipotent until the formation of the blastocyst. At this stage, the inner cell mass begins to differentiate, and the cells are thereafter considered pluripotent [2].

Pluripotent cells can give rise to cells belonging to any of the three germ layers (endoderm, mesoderm, ectoderm) but lack the ability to differentiate into extraembryonic cells. Upon further maturation and differentiation, stem cells change from pluripotent to multipotent. Although multipotent cells are still capable of differentiating into a small number of discrete cell types, they are limited to cell types that are related to one another (e.g., neural stem cells, mesenchymal stem cells).

In vivo stem cells can be broadly divided into three types based on their origin: embryonic (ESCs), fetal (FSCs), and adult stem cells (ASCs, among them mesenchymal stem cells, or MSCs).

Embryonic stem cells (ESCs) are derived from the inner cell mass on the pre-implantation embryo, known as a blastocyst, post-fertilization. These pluripotent cells, found in the blastocyst’s inner cell mass, multiply and differentiate to different cell types constituting the organism.

Fetal stem cells (FSCs) are multipotent cells located in fetal tissues [3]. Depending on the tissues they are capable of generating, they can be further divided into hematopoietic (giving rise to blood, liver, and bone marrow-associated cell types), mesenchymal (which can generate blood, liver, bone marrow, lung, kidney, and pancreatic cells), endothelial (capable of differentiating into bone marrow and placental cells), epithelial (giving rise to liver and pancreatic cells), and neural stem cells (which differentiate into brain and spinal cord tissues).

Adult stem cells (ASCs) are multipotent cells that are found in various tissues throughout the body. Also called somatic stem cells, they can be found in both juvenile and adult animals and humans, unlike ESCs and FSCs. Although ASCs can be found in most tissues throughout the body, the most common sources of autologous ASCs in humans are bone marrow and adipose tissue. ASCs tend to be lineage-restricted (multipotent) in terms of their differentiation potential, and they, like FSCs, are typically referred to by their tissue of origin (e.g., mesenchymal stem cells, hematopoietic stem cells, neural stem cells).

Another rich source of stem cells in humans is umbilical cord blood. These multipotent stem cells can be found in both the cord blood and tissue (Wharton’s jelly), and they, along with FSCs, are referred to as perinatal stem cells. Given the therapeutic potential of the stem cell platform, cord blood banking is increasingly being touted as a viable option to treat disorders that may arise later in a person’s life, with cord blood also being used in transplantation to overcome human leukocyte antigen (HLA) incompatibility [4].

**Figure 2:**
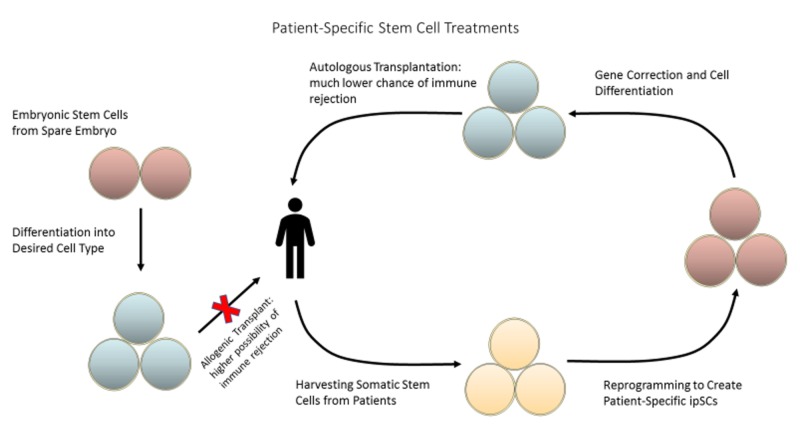
Somatic stem cells can be harvested from the patient and reprogrammed into induced pluripotent stem cells (ipSCs) to create patient-specific therapies, reducing the risk of immune rejection

Although multipotent adult stem cells are limited in their ability to differentiate, methods have been developed to overcome these hurdles. Induced pluripotent stem cells (iPSCs) are adult cells that have been artificially reprogrammed to behave like embryonic stem cells, as shown in Figure 2. By introducing a specific set of transcription factors (Oct3/4, Sox2, Klf4, and c-Myc), somatic cells are able to regain their pluripotency, thus allotting them tremendous therapeutic potential.

## Review

Naïve stem cells as therapeutic missiles for various diseases

Recent advances in genetic engineering have allotted us the ability to alter cells, most notably, stem cells, in ways that allow for very specific and effective targeted functionalities. Despite this, unmodified stem cells still possess a great therapeutic potential and are frequently used in modern therapies.

Regenerative Medicine

As a result of the recent advances in stem cell technologies, we are now able to study the mechanisms of human disease more effectively. This has ushered in a new era for regenerative medicine, extending beyond early cell-based therapies and moving towards evaluating genetic variation in humans and identifying the molecular pathways that lead to disease, as well as targets for therapy [5].

Ischemic Cardiomyopathy

Cardiovascular disease remains the leading cause of death in the United States. Over the last decade, stem cells have emerged as a potential therapeutic agent for chronically injured tissue, with MSCs being widely studied for such therapies. Preliminary results have shown promising outcomes in the repair and generation of cardiac tissue [6].

A double-blind, placebo-controlled study used intravenous allogeneic human MSCs in 53 post-MI patients, with similar adverse events between both groups. Ambulatory EKG showed reduced episodes of ventricular tachycardia, as well as improved forced expiratory volume in one second on pulmonary function testing in the group that received MSC transfusion. Global symptoms score and ejection fraction were both significantly improved in the MSC group [7]. Similar results were seen in another study, where MSC injection led to improved functional capacity, quality of life, and ventricular remodeling in patients with ischemic cardiomyopathy [8].

Musculoskeletal Disorders

Stem cell therapies have also offered new prospects for the treatment of musculoskeletal diseases. Various factors can be modified to promote the differentiation and growth of stem cells into complex tissues composed of different cell types due to their multilineage differentiation capabilities. Various techniques have been developed to create the appropriate microenvironment for the stem cells to modulate their behavior and fate. This includes the composition of the scaffolding, growth factors, and oxygen tension [9]. Stem-cell-based therapies are also being vigorously studied in therapies for maxillofacial disorders/defects. There are at least 44 ongoing registered clinical trials related to oral stem cells and oral disease [10]. Stem cell therapies can be designed to target different stages of osteoarthritis. Early-stage treatments focus on the manipulation of endogenous stem cells and intra-articular stem cell injection, while treatments in later stages focus on joint resurfacing. The main goal of these treatments is to initiate chondrogenesis. Some notable ongoing clinical trials are utilizing autologous stem cells from the bone marrow and transplanting them into the joint space. Another trial that was published studied 13 patients, with a mean age of 50 years, who were given intra-articular stem cell injections. MRI scans on serial follow-ups showed improvement in the health and quality of the treated articular cartilage [11].

Retinal Disorders

Degenerative retinal diseases are some of the most challenging disorders to treat for ophthalmologists. The lack of reliable options often means that patients receive inadequate treatment for these debilitating illnesses. Stem cells are currently being investigated as a treatment for retinal disorders. Their differentiation and self-renewal capabilities indicate that they have the potential to spawn novel, more effective treatments for retinal disorders, and maybe reverse blindness in the future. After an initial study showed promising results, a second stem-cell-based ophthalmology treatment study was initiated and it is currently ongoing. The suggested model through which the stem cells are working includes the migration of the stem cells to the injured areas, and their differentiation into retinal pigment cells, rather than releasing neurotrophic growth factors [12].

Autoimmune Diseases

It has been demonstrated by multiple studies that stem cells demonstrate immunoregulatory properties based on their interactions with the cellular components of both innate and adaptive immune systems.

Most cases of type 1 diabetes are caused by a T-cell-mediated autoimmune attack, which results in the destruction of β-cells and thus the ability to produce insulin naturally. This renders the patient dependent upon exogenous insulin for the rest of their life. Several studies have achieved some success by aiming to replace lost β-cells via stem-cell-based therapies. A recent study compared iPSC and MSC transplantation in an animal model of insulin-dependent diabetes. This study showed that iPSC transplantation initiated endogenous pancreatic regeneration by neogenesis of islets and concluded that iPSC-based therapies can offer hope to patients with type 1 diabetes [13].

Although β-cell replacement may be effective, there are concerns that the same autoimmune response that initially destroyed the patient’s β-cells could simply occur again. Another approach, called “stem cell educator therapy,” has been developed to modulate the patient’s autoimmune response by employing stem cell therapy, and it has produced promising results. The patient’s blood was circulated through a closed-loop system to separate their lymphocytes. These are then co-cultured with human cord blood-derived multipotent stem cells and returned to the patient. Initial results indicated that the islet cell-prompted autoimmune response was reversed and the regeneration of islet cells was observed [14].

For rheumatoid arthritis, a comparison between disease-modifying antirheumatic drugs (DMARDs) plus medium without stems cells and DMARDs plus umbilical cord mesenchymal stem cell (UC-MSC) given intravenously showed no serious adverse effects, and serum levels of TNF-alpha and IL-6 were observed to have decreased after the first treatment. The stem-cell-based treatment induced significant remission of the disease as measured by American College of Rheumatology’s improvement criteria, the 28 joint disease activity score and Health Assessment Questionnaire, while the control group displayed no such benefits [15]. A review of studies looking into autologous hematopoietic stem cell transplantation (HSCT) in SLE and antiphospholipid syndrome evaluated 25 studies. Most of the studies reported an improvement after HSCT when compared to immunosuppression alone, as assessed with systemic lupus erythematosus disease activity index (SLEDAI) or disease-free time [16].

Multiple sclerosis, a demyelinating disease characterized by a progressive decline in function, is a devastating disease for patients but one to which the stem cell platform can be applied. A recent trial enrolled 20 subjects who were given umbilical cord-derived mesenchymal stem cells as treatment, and they were followed up for one year. The patients showed a statistically significant improvement in their EDSS scores (p<0.03) [17].

ALS is another debilitating disease that can benefit from the use of stem cell-based therapies. So far, two different approaches have been utilized to treat this disease: one approach is to regenerate motor neurons using stem cells and replace the cells lost as the disease progresses; another approach is to use stem cells to regenerate new astrocytes to replace the dysfunctional cells that are responsible for the toxic environment that contributes to the death of neurons. These trials, most of which are administering stem cells intrathecally, are utilizing hematopoietic, mesenchymal, and neuronal stem cells to achieve their goal [18].

Engineered stem cells

The ability to genetically engineer stem cells has paved the way for countless therapeutic opportunities in modern medicine. Despite the great potential of the stem cell platform, the field in which this potential is being fully explored is oncology. Stem cells can be engineered to enhance their homing towards cancer cells, act as carriers of bioactive materials, or secrete immune-modulators that enhance the immune system’s ability to eradicate tumor cells.

Engineered Cells with Anti-Cancer Activity

Initial studies showed that treatment with bone marrow-derived MSCs genetically engineered to secrete IFN-β caused them to incorporate into malignant tissue, while locally secreting IFN-β and inhibiting tumor growth in a human melanoma mouse xenotransplantation model. This significant effect is not attainable through the systemic administration of IFN-β. In a later study involving a canine melanoma model, it was also shown that IFN-β-transduced adipose tissue-derived MSCs can be used as carriers of anti-tumor prodrugs, in this case, cisplatin, which improved therapeutic efficacy [19].

Many other cytokines and tumor suppressor genes with anticancer activities are currently being used in the genetic modification of MSCs. These therapies aim to selectively suppress and eliminate tumor cells, increasing the efficacy of the therapies and reducing toxicity. One of the most promising of these is tumor necrosis factor-related apoptosis-inducing ligand (TRAIL). TRAIL is a type II transmembrane protein that allows for selective triggering apoptosis in tumor cells while sparing normal cells. Despite TRAIL’s great potential in cancer treatments, there are many challenges in developing TRAIL-based therapies, namely, its short half-life of approximately 30 minutes, limited bioavailability, and poor pharmacokinetics. One study has shown that TRAIL’s short expression can be overcome by IFN-γ-secreting MSCs [20], which led to the long-term expression of TRAIL while another study showed that combining recombinant TNF-alpha-activated MSCs coupled with radiation exposure can increase therapeutic efficacy [21].

MSCs can serve as useful delivery vehicles for IL-12 and other antineoplastic agents in brain tumor therapies. The peripheral administration of human MSCs transduced with a recombinant adenoviral vector expressing murine IL-12 in a nude model of renal cell carcinoma in mice showed the homing of these IL-12-expressing MSCs to the tumor cells. These cells secreted IL-12 locally, with only a modest increase in systemic IL-12 levels, thus demonstrating the potential of adult MSCs administering IL-12 to reduce tumor growth and enhance survival [22].

MSCs can also be transfected with anticancer genes like PTEN (phosphatase and tensin homolog, deleted on chromosome 10). MSCs retain their tumor-homing properties after undergoing genetic engineering, and PTEN-modified MSCs have been shown to exhibit anti-cancer effects against glioma cells in vitro [23].

The homing properties of stem cells can also be used to transport anti-cancer drugs directly to tumor cells. Pessina et al. (2013) showed the concentration of paclitaxel (PTX) necessary to decrease the viability of certain types of tumor cells without adversely affecting the MSCs [24]. The same study demonstrated that PTX-primed MSCs inhibited the growth of tumor cells as well as inhibited tumor vascularization in a leukemia xenograft mouse model.

Engineered Stem Cells in Neurological Disorders

The main goal of stem cell therapies in neurological disorders is to replace damaged and non-functional tissues, improving neurological outcomes. However, the efficacy of stem cell-based therapies is greatly affected by the microenvironment found in the target tissue. To overcome the processes impeding stem cell integration into the tissue, stem cells are engineered to secrete and deliver molecules that can enhance differentiation and vascularization, improving the outcomes of stem cell-based therapies [25].

Spinal Cord Injuries

In spinal cord injuries (SCI), there have been some promising outcomes utilizing NSC transplantation, with the underlying mechanism not being completely understood. Transplantation of naïve NSCs has been shown to improve motor function in SCI, with aberrant host fiber sprouting also being seen in rodent models associated with allodynia-like hypersensitivity. One can conclude that the controlled differentiation of these stem cells via stem cell engineering is an essential part of the process to avoid side effects and observe significant improvements in function. One way of achieving this is by engineering NSCs to secrete neurotrophins. These are a family of growth factors that can promote the survival, development, and function of neurons. Grill et al. demonstrated that primary skin fibroblasts can be engineered to secrete neurotrophin-3 (NT-3), which, when transplanted into SCI models, promoted corticospinal tract regeneration and motor function improvement. NSCs that have been genetically engineered to secrete higher levels of NT-3 could greatly enhance the therapeutic effects of this therapy when compared to naïve NSCs [26].

Controlling the differentiation of these cells is important to maximize their therapeutic benefits. OLIG is a family of transcription factors that are believed to be key regulators of oligodendrocyte-specific differentiation during development. To further enhance the differentiation of NSCs into oligodendrocytes, engineered NSCs to express Olig2 can be combined with myelin basic protein-activated T cells [27]. The MBP-T cells infiltrate the transplant site, modulating the local microenvironment and changing the inflammatory response of local T cells, as well as the microglial response. This also leads to an increase in brain-derived neurotrophic factors, as well as the differentiation of resident microglia and infiltrating blood monocytes into “alternatively activated” anti-inflammatory macrophages. As a result, newly formed neurons were observed from the endogenous NSC pool.

Parkinson’s Disease

Besides medical therapy, various strategies have been suggested for achieving long-term treatment of Parkinson’s. GTPCH1 is a key enzyme in the synthesis of tetrahydrobiopterin, a cofactor that supports TH activity. Human NSCs have since been engineered to secrete a combination of levodopa (L-DOPA), tyrosine hydroxylase (TH), and guanosine 5'-triphosphate (GTP) cyclohydrolase 1 (GTHCH1) using transduction with a retroviral vector encoding TH and GTPCH1 [28]. The amount of L-DOPA produced by the engineered cells was significantly higher than the unengineered NSCs or NSCs transduced with only the TH gene. These engineered stem cells were then transplanted into the striata of hemiparkinsonian rats. These cells did not face any rejection and survived well in the host brain. Though some of the cells migrated to sites away from the injection site, they maintained high-level TH expression for up to four weeks. Functional improvements were also seen, suggesting that engineered NSCs expressing both TH and GTPCH1 could have great potential for the treatment of Parkinson’s and should be explored further.

Other molecules also play a role in the pathophysiology of Parkinson’s, and their incorporation into stem cell-based therapies may improve outcomes. Nuclear receptor-related 1 protein (NURR1) is a member of the steroid/thyroid hormone orphan nuclear receptor family and an important factor expressed in dopaminergic neurons. Brn4 is a member of the POU-homeodomain family and plays a role in neuronal migration and differentiation. Engineering NSCs with both NURR1 and Brn4 could dramatically increase the differentiation and maturity of TH-expressing dopaminergic neurons [29]. These NSCs, when transplanted in rat Parkinson’s model, showed that the overexpression of NURR1 alone was able to promote the differentiation of NSCs into dopaminergic neurons in vivo, resulting in increased levels of dopamine in the striatum. Co-expression of both NURR1 and Brn1 significantly increased the viability and maturity of these dopaminergic neurons, with behavioral improvements being seen as well.

Stroke

Most modern therapies for stroke involve thrombolysis, reducing the risk of recurrent stroke and the rehabilitation of the patients. However, no therapies are available for the restoration of the damaged neural tissue and the restoration of function. The transplantation of MSCs post-stroke has resulted in observable improvements in functional recovery, and these stem cells can be engineered to further their neuro-regenerative and neuroprotective properties. Several molecules have been screened for this purpose. One of them is the hepatocyte growth factor (HGF). HGF plays a role in anti-apoptosis, angiogenesis, motogenesis, morphogenesis, tissue regeneration, and neurite outgrowth enhancement, in addition to the inhibition of blood-brain barrier (BBB) destruction. MSCs engineered with HGF, via a multimutated HSC-1 vector (MSC-HGF), were used to treat brain ischemia in the superacute and acute therapeutic phases in a transient middle cerebral artery occlusion model in rats. They showed significant improvements in the neurological deficit as compared to MSCs alone [30].

One other molecule that has been studied is Akt1, which has been demonstrated to promote cell survival during free radical exposure or hypoxia in hippocampal neurons. NSCs engineered with Akt1 were highly resistant to hydrogen peroxide (H2O2)-induced cytotoxicity in vitro. Post-transplantation in the stroke mouse model, behavioral improvement and significantly increased cell survival were seen [31].

Another molecule is angiopoietin-1 (Ang-1), known for its role in promoting angiogenesis in brain tissue. It is also known to protect the peripheral vasculature from leakage, which may be the mechanism for its role in preventing edema following ischemia. MSCs engineered with Ang-1 could potentially enhance functional recovery post-ischemic event by improving angiogenesis in the tissue surrounding ischemic lesions [32]. In a rat middle cerebral artery occlusion model, they were infused with MSCs and Ang-MSCs 6 hours post-stroke. Both a reduction in infarction volume and an improvement in function were noted.

Challenges

Although stem cell-based therapies can be used to effectively treat a myriad of human disorders, they are not without their challenges.

Tumorigenicity of Stem Cells

Stem cells are defined by their ability to self-renew and differentiate into various cell types: the properties that make them ideal for the potential replacement of diseased or damaged tissue. However, these same properties are responsible for their tumorigenic potential. The risk of tumorigenicity in the stem cell platform has been highlighted in many studies, including preclinical dose-escalation tests for the first-in-human induced pluripotent stem cells (iPSC) clinical trial to be approved by the Food and Drug Administration (FDA) in 2009. Cysts in the regenerating spinal tissue were seen in mice that received human ESC-derived neural progenitor cells, leading to a one-year moratorium on the study before any human subject received the treatment [33]. In humans, there are several case reports of tumors in patients who received stem-cell-based therapies, as evidenced by tumor formation four years after fetal neural stem cell transplantation for ataxia-telangiectasia in a 12-year-old child [34]. Another case reported a tumor in a 46-year-old woman who had received autologous hematopoietic stem cell transplantation for the treatment of lupus nephritis [35].

Treatment Durability

Even though targeted stem cell therapies for cancers have a strong therapeutic effect initially, relapse commonly occurs. Therefore, stem cell-based therapies alone are likely not enough to eradicate tumors. To achieve the greatest therapeutic benefit for the patient, these targeted therapies are often combined with other rationally selected therapies. Two ongoing clinical trials are looking into the role of NSCs in human Glioblastoma multiforme (GBM), and the outcomes will provide exciting clinical feedback [36]. Several other stem cell combination therapies have also been studied. In one study, oncolytic virus-loaded NSCs were used to infect GBM tumor cells prior to ionizing radiation (XRT) and temozolomide (TMZ) treatment. The introduction of the oncolytic virus (OV)-loaded NSCs increased the tumor cells’ radiosensitivity and resulted in a 30% increase in survival in a glioma xenograft model [37].

Ethical Considerations

Human embryonic stem cells (hESCs) were isolated for the first time in 1998, from an embryo donated by a couple who no longer wished to use it for their infertility treatment. Since then, hESC research has been a subject of controversy, which is part of society’s underlying fears about the reach of science, involving human cloning and commodification of human biological material. However, most of the concerns surrounding the use of hESCs in research stem from the destruction of human embryos [38].

To mitigate these ethical concerns, alternative approaches have been proposed. One approach is a variation of somatic cell nuclear transfer (SCNT), in which the deoxyribonucleic acid (DNA) of an unfertilized egg is replaced by the nucleus of a somatic cell. A genetic defect is introduced into the nucleus of the somatic cell prior to the transfer, which blocks the implantation of the developing embryo. Thus, a cloned mouse embryo was created that generated pluripotent stem cells before arresting developmentally. If a similar technique were used with a human somatic cell and an unfertilized egg, this would potentially result in a blastocyst that could produce pluripotent stem cells but would lack the ability to develop into a complete human being. It was suggested that these altered nuclear transfer (ANT) products should be considered complex tissue cultures rather than viable human embryos [39]. However, there are still numerous uncertainties surrounding ANT in human cells, as concluded by the study. For example, it remains uncertain whether the CDX2-deficient human embryo will behave the same way as CDX2-deficient mouse embryos. Similarly, the manipulation of somatic cell DNA may give rise to safety concerns in patient-specific stem cell lines.

Fortunately, the advent of iPSCs has alleviated most ethical concerns regarding stem cell research involving hESCs. Though research involving iPSCs comes with its own set of ethical concerns, such as the donor’s consent for the extent of use of their genetically matched iPSC lines, as well as what to do in case of incidental findings that may impact the donor’s health [40].

However, SCNT faces many hurdles that stop it from being a widespread methodology in stem cell research. NIH guidelines allow for the use of federal funding for excess IVF embryos but not for embryos created specifically for research. The other issue is that women are not willing to donate their eggs without compensation. In some states, it is illegal to compensate women for donating their eggs, and the practice is not recommended by the National Academy of Sciences’ Guidelines for Human Embryonic Stem Cell Research.

As of late, a new system of research oversight in stem cell research has evolved. The National Academies and the International Society for Stem Cell Research (ISSCR) encourage all researchers working with pluripotent stem cells to have their research approved by the Stem Cell Research Oversight Committee (SCRO). SCRO consists of scientists, physicians, ethicists, legal experts, and even community members. SCRO ensures that local ethical requirements are met, voluntary informed consent is obtained from donors, and donors of human materials, including embryos, are treated fairly.

Cost

**Figure 3:**
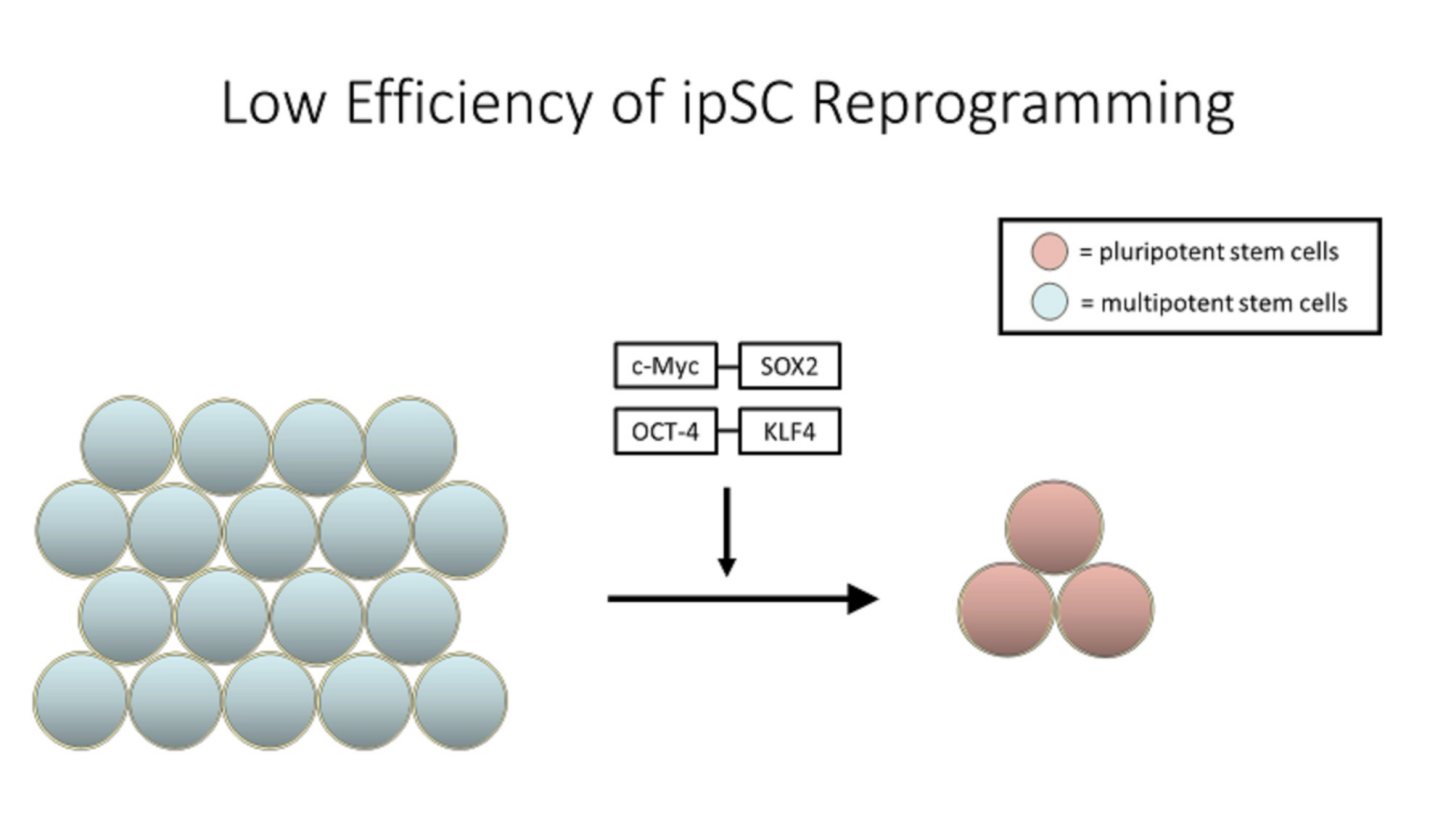
Each cell line of human iPSCs requires two to four months to develop, starting with the collection of primary cells, which are then reprogrammed, with efficiencies of approximately 0.01% to 0.1%, and grown into a sizable induced pluripotent stem cell (iPSC) population.

**Figure 4:**
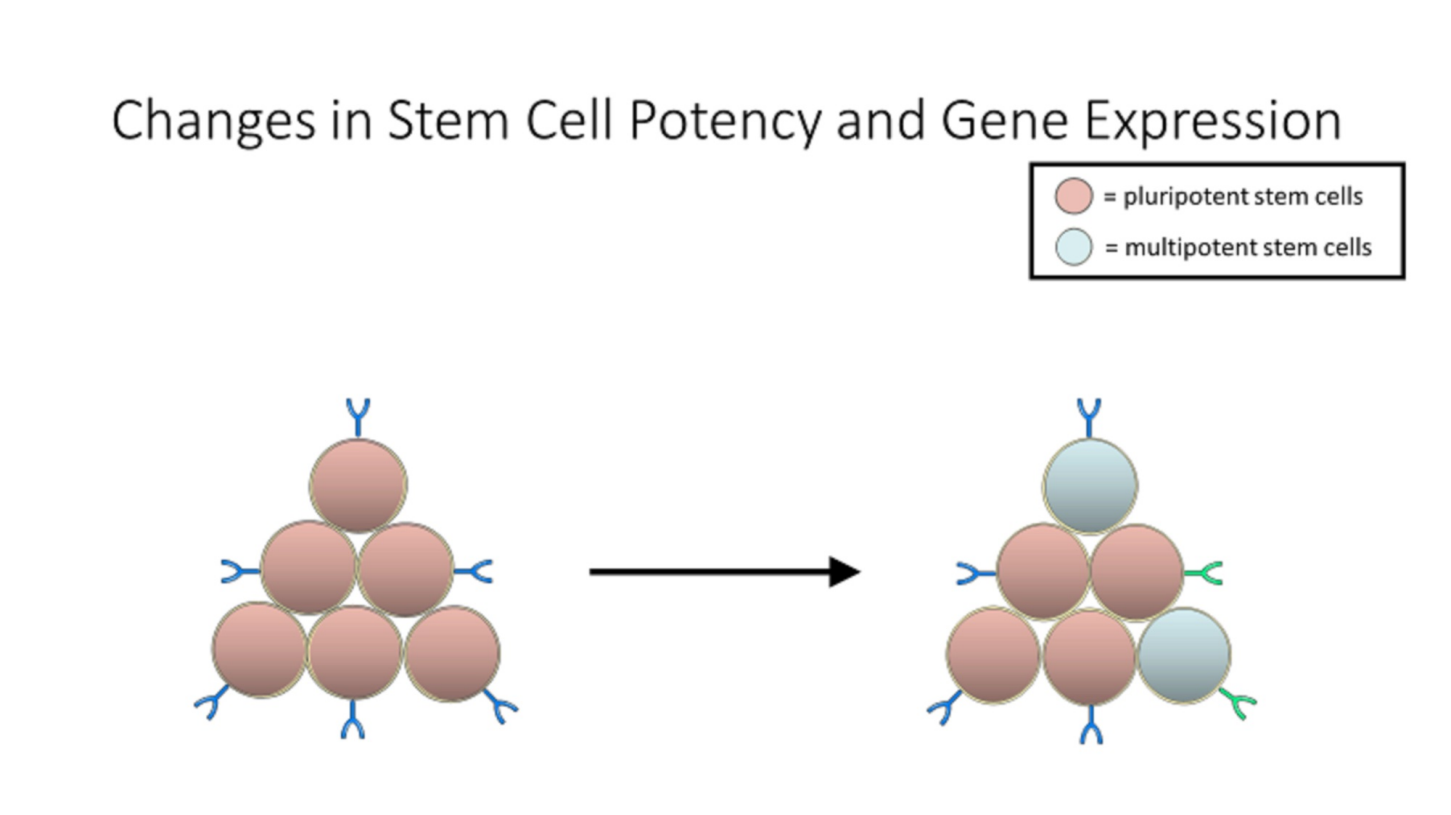
Keeping cells in culture for the long periods of time required to reprogram induced pluripotent stem cells (iPSCs) can also result in changes in potency in gene expression. This can result in cells that are not viable for therapeutic purposes, and those will have to be sorted through.

One of the largest hurdles standing in the way of the mainstream availability of stem cell-based therapies and research is cost. Each cell line of human iPSCs requires two to four months to develop, starting with the collection of primary cells, which are then reprogrammed, with efficiencies of approximately 0.01% to 0.1% (Figure 3), and grown into a sizable iPSC population [41]. Furthermore, keeping cells in culture for the long periods of time required to reprogram iPSCs can also result in changes in potency in gene expression (Figure 4). This can result in cells that are not viable for therapeutic purposes, and those will have to be sorted through. As a result, developing clinical-grade iPSC-derived tissue products is exorbitantly expensive (~$800,000) and considering the uncertainties and lack of clinical data involved with many of these therapies, one may argue that these therapies are not attainable or accessible [42]. Certain strategies have been suggested to reduce the cost of these therapies, one of which is to test multiple cell lines together without compromising safety or efficacy. This is especially cost-effective in case of iPSC lines, as, generally speaking, three cell lines are derived from a single donor [43].

## Conclusions

Stem cells, due to their regenerative, transformative, and homing properties, theoretically have the potential to cure any condition that involves cellular pathology by replacing those cells. Not only do they hold the power to treat cancer more effectively, but they can also help patients suffering from chronic conditions such as strokes, dementia, Parkinson’s, and diabetes, as well as postulate the possibility of curing conditions long thought to be incurable. However, a lot of these breakthroughs are in animal models or in vitro and may not have clinically significant therapeutic effects in human models. Similarly, various transformation pathways of MSCs are not completely understood, and concerns for the spontaneous development of malignancies are somewhat valid. A search on clinicaltrials.gov reveals more than 500 ongoing clinical trials involving stem cell-based treatment, but we are far from many of these therapies being available for widespread clinical adaptation. Even though stem-cells-based therapies come with their set of challenges and concerns, and they necessitate the need for combination therapies, it is evident that stem cells are the way forward, and they should be treated as such.
